# Effect of *Claroideoglomous etunicatums* on rhizosphere bacterial community of tobacco under low nutrient conditions

**DOI:** 10.1007/s44307-025-00071-x

**Published:** 2025-07-04

**Authors:** Jin Chen, Xiaowan Geng, Qing Zhang, Keqing Lin, Zishan Li, Boyan Wang, Qingchen Xiao, Xiaoyu Li

**Affiliations:** 1 Schools of Life Sciences, Anhui Agricultural University, Hefei, 230036 China; 2 National Engineering Laboratory of Crop Stress Resistance Breeding, Anhui Agricultural University, Hefei, 230036 China; 3 Key Laboratory of Crop Stress Resistance and High-Quality Biology of Anhui Province, Anhui Agricultural University, Hefei, 230036 China

**Keywords:** Arbuscular mycorrhizal fungi, Tobacco, *Claroideoglomous etunicatums*, Bacterial community, Illumina MiSeq high-throughput sequencing

## Abstract

Arbuscular mycorrhizal fungi (AMF) have the potential to enhance plant tolerance to abiotic stresses. However, the impact of AMF on the rhizosphere bacterial community of tobacco under conditions of low nutrient availability remains unclear. This study investigated the influence of inoculating *Claroideoglomus etunicatum* on the tobacco rhizosphere bacterial community and the microbial mechanisms by which AMF enhanced plants antioxidant capacity, employing Illumina MiSeq high-throughput sequencing. The findings indicated that AMF significantly increased both the aboveground and belowground fresh weight, as well as the plant height of tobacco. AMF inoculation led to elevated activities of catalase (CAT) and superoxide dismutase (SOD), a reduction in malondialdehyde (MDA) content, and an overall enhancement of the plants antioxidant capacity. Phylogenetic analysis demonstrated that AMF modified the bacterial community structure and significantly enriched beneficial rhizosphere bacteria, predominantly from the phyla Proteobacteria, Chloroflexi, Actinobacteriota, and Myxococcota, thereby facilitating tobacco growth. The network analysis revealed that the incorporation of arbuscular mycorrhizal fungi (AMF) contributed to increased stability within the bacterial community, enriched species diversity, and more intricate ecological networks. AMF enhanced interactions and positive correlations among bacterial species, indicating that heightened microbial synergy is associated with improved symbiotic relationships. Furthermore, the structural equation model demonstrated that AMF bolstered the plants antioxidant capacity by modulating the rhizosphere bacterial community. This study elucidates the impact of AMF on the tobacco rhizosphere bacterial community, providing a theoretical basis for promoting tobacco growth.

## Introduction

Arbuscular mycorrhizal fungi (AMF) can establish symbiotic relationships with approximately 80% of plant species, including most agricultural crops, and are prevalent in terrestrial ecosystems. Through their extraradical mycelium, AMF improved water and nutrient absorption in host plants under stress conditions and stimulate plant growth by facilitating the uptake of limiting nutrients, such as phosphorus, from the soil (Zhang et al. 2022a, b; Cao et al. 2024). Enhancing plant tolerance and maintaining crop productivity under low-nutrient conditions poses a significant challenge for sustainable agriculture. In response to abiotic stresses, many plants evolve a variety of defense mechanisms. Among these strategies, the utilization of beneficial microorganisms, such as AMF, has demonstrated the potential to support host plants through root symbiotic associations (Alsherif et al. 2023; Zhao et al. 2025). AMF species belonged to the Glomeromycotina subphylum of Mucoromycota (Abkenar et al. 2024). AMF is generally recognized for its role in improving plant tolerance to abiotic stress and enhancing the capacity of plants to remediate soil. Numerous studies have indicated that AMF mitigates the adverse effects of various stresses by enhancing nutrient absorption, particularly phosphorus (P), bolstering the antioxidant system, preserving enzyme activity, and altering the composition of the rhizosphere microbial community to reduce damage induced by abiotic stress (Riaz et al. 2021; Bilgili and Bilgili., 2023). Previous research has established that AMF could alleviate the harm caused by abiotic stress. This was achieved through the enhancement of plant mineral nutrient uptake via the formation of an extensive mycelial network (Chen et al. 2024b; Zhao et al. 2025). AMF enhanced plant mineral nutrient uptake by establishing an extensive mycelial network. The extraradical hyphae of AMF absorbed soil mineral nutrients and facilitated their transport to the plant roots and throughout the plant system. Additionally, AMF acquired organic carbon from plants via arbuscular symbiotic structures (Gong et al. 2022). Consequently, it is of significant importance to investigate the alterations in the microbial community diversity within the tobacco rhizosphere under low-nutrient conditions and to elucidate the impact of AMF on the microbial community structure during the growth of tobacco.

Soil microorganisms were pivotal in influencing the sustainability of soil resources and ecosystem functions, playing crucial roles in soil nutrient cycling, organic matter decomposition, soil remediation, and plant growth (Li et al. 2024; Ding et al. 2024). The rhizosphere, defined as the soil region adjacent to plant roots, represented the most active microhabitat for microbial interactions (Afridi et al. 2022). Given the critical role of rhizosphere microorganisms in plant growth and health, there has been an increasing focus on understanding the interactions between plants and rhizosphere microbial communities. Alterations in the structure of the rhizosphere microbial community have been shown to impact plant root growth and soil nutrient cycling (Cui et al. 2023). Additionally, research indicated that specific microorganisms, particularly rhizosphere bacteria, contribute to plant growth enhancement and increased stress tolerance by stimulating soil enzyme activity (Liu et al. 2023a, b). Rhizosphere bacteria, as the principal microorganisms within plant rhizospheres, exhibited high sensitivity to environmental changes and were integral to complex biological and ecological processes, thereby played a vital role in plant growth and phytoremediation efficiency (Liu et al. 2024). Several growth-promoting bacteria in the plant rhizosphere facilitated root development and elongation, as well as improved plant nutrient acquisition by engaging in plant hormone-mediated signal transduction mechanisms (Wang et al. 2022). Mycorrhizal fungi, including arbuscular mycorrhizal and ectomycorrhizal fungi, increased soil nutrient availability through symbiotic associations with plant roots (Duan, et al. 2024). Furthermore, mycorrhizal fungi, including both arbuscular and ectomycorrhizal types, enhanced soil nutrient availability through their symbiotic associations with plant roots (Timofeeva et al. 2024). In contrast, due to long-term evolutionary processes and environmental adaptations, plant roots exude a variety of compounds and specific molecules that facilitate nutrient cycling and influence the composition of the rhizosphere microbiota. Thus, understanding plant-microorganism interactions and implementing effective strategies to manage crop rhizosphere microecology are crucial for achieving high-quality and sustainable crop production. Despite this importance, research on the effects of arbuscular mycorrhizal fungi (AMF) on tobacco rhizosphere bacterial communities under low-nutrient conditions remains limited.

Tobacco has historically been a crop of significant economic importance (Jiang et al. 2024). In particular, within the context of China, tobacco hold substantial economic value. It was one of the most extensively researched plants and is known for its ability to form symbiotic relationships with AMF (Li et al. 2021a; Li et al. 2023a, b). In this study, tobacco was cultivated in a controlled greenhouse pot experiment using loess soil. The primary objectives of this research were to: (1) examine the impact of AMF on tobacco growth and the structure of the rhizosphere bacterial community under conditions of low nutrient availability, and (2) assess the influence of AMF inoculation on the diversity, abundance, and composition of the rhizosphere bacterial community associated with tobacco.

## Materials and method

### Soil preparation

The soil used in the pot experiment was sourced from yellow cinnamon soils (Chromic-Leptic Luvisols in the WRB) at Anhui Agricultural University (31°93′N, 117°21′E), Anhui, China. The soil properties were characterized as follows: ammonium nitrogen content of 3.37 mg kg⁻^1^, available phosphorus content of 42.7 mg kg⁻^1^, nitrate nitrogen content of 2.16 mg kg⁻^1^, fast-acting potassium content of 156 mg kg⁻^1^, soil organic matter content of 15.3 g kg⁻^1^, total carbon content of 10.13 g kg⁻^1^, total phosphorus content of 0.50 g kg⁻^1^, total potassium content of 21.13 g kg⁻^1^, and total nitrogen content of 0.80 g kg⁻^1^. Subsequently, the fresh soil was dried and sieved through a 2 mm mesh. The pot experiment was conducted using 17.7 × 12 cm (diameter × height) pots containing 1 kg of mixed vermiculite substrate (1:1, w/w). To eliminate AMF and other microorganisms, the soil was autoclaved at 121 °C for 2 h in batches of approximately 18 kg each.

### Fungal and plant material

In this study, the AMF strain utilized was *Claroideoglomus etunicatum* (BGC NM01B, 1511 C0001BGCAM0017), while tobacco (*Nicotiana tabacum* L.) seeds were stored at the State-Local Joint Engineering Laboratory of Crop Resistance Breeding and Disaster Reduction, Anhui Agricultural University. The inoculum consisted of sand, spores, mycelia, and colonized root fragments. The AMF was propagated in sandy soil through a pot experiment using maize. The experiment was conducted at the nursery garden of Anhui Agricultural University. The seeds were surface-sterilized using 10% hydrogen peroxide (H_2_O_2_) for 10 min and subsequently rinsed thoroughly with sterile water. Sterilized seeds were placed in sterile black containers filled with an autoclaved mixture of vermiculite and nutrient soil in a 3:1 volume-to-volume ratio. After one week in the incubator, seedlings exhibiting robust growth were selected for the pot experiment.

### Experimental design and sample collection

Plants were cultivated in greenhouses at the nursery facility of Anhui Agricultural University from May 10 to July 10, 2021, under controlled temperature conditions of 28/18 °C (light/nigh). The experimental design was a completely randomized design with three biological replicates for both the CK and AMF treatments. Each pot received an inoculation of 30 g (containing 5 spores per 1 g). For the uninoculated control, an equivalent amount of autoclaved inoculum was incorporated into the soil to maintain consistent microbial populations across different treatments. Throughout the experiment, plants were consistently watered.

After 60 days, the rhizosphere soil from both the CK and AMF treatment groups was meticulously separated from the roots using the method outlined by Edwards et al. (2015). Subsequently, under sterile conditions, soil adhering to the plant roots was rinsed with sterile water. The soil was then centrifuged at high speed (4 °C, 8000 rpm) for 10 min and thoroughly mixed to form a composite sample. A portion of this sample was stored at 4 °C for subsequent analysis of soil spore density and enzyme activity, while another portion was stored at −80 °C for high-throughput sequencing. The remaining soil was subjected to drying for the analysis of selected physical and chemical properties.

### Determination of Root Mycorrhizal Colonization

Segments of tobacco roots, each measuring 1 cm, were immersed in 10% KOH solution, heated at 90 °C for 1 h. Subsequently, the roots were rinsed with distilled water to eliminate any residual KOH, immersed in a 5% lactic acid solution for five minutes, and stained with a 0.05% trypan blue solution. Following the removal of excess dye, the roots were soaked in a lactic acid-glycerol solution and placed on a shaker to facilitate decolorization. The roots were then sectioned and mounted on microscope slides (Li et al. 2023a, b). The rate of mycorrhizal colonization was quantified as the percentage of AMF-infected root length relative to the total observed root length.

### Determination of antioxidant enzymes activities and MDA

Enzyme activities were quantified employing the methodologies outlined by Gao (2006). Specifically, catalase (CAT) and peroxidase (POD) activities were evaluated by measuring the absorbance of the reaction mixtures at wavelengths of 470 nm and 240 nm, respectively, using a UV/visible spectrophotometer. Fresh leaf samples, weighing 0.5 g, were homogenized and subjected to centrifugation as described by Zhang (2023). The absorbance of the reaction mixture was subsequently measured at 510 nm with a UV/visible spectrophotometer (UV-560B, METASH, China) to ascertain the respective values. Malondialdehyde (MDA) content was determined following the procedure of Gao (2006). Fresh leaf tissue samples (0.5 g) were pulverized in a mortar, and the absorbance values of the supernatant was recorded at 532, 600, and 450 nm using a UV/visible spectrophotometer.

### Illumina sequencing of rhizosphere bacteria

In alignment with the manufacturer's protocol, three replicate rhizosphere soil samples (each weighing 0.4 g) were extracted using the Power Soil DNA Isolation Kit (MO BIO Laboratory, Carlsbad, USA). Sequencing was performed by Majorbio (Shanghai, China) on the Illumina MiSeq PE300 platform, following the manufacturer's instructions. The concentration and purity of DNA were evaluated using a NanoDrop 2000 UV–vis spectrophotometer (Thermo Scientific, Wilmington, USA). Primers 338 F (5′-ACTCCTACGGGAGGCAGCA-3′) and 806 R (5′-GGACTACHV-GGGTWTCTAAT-3′) amplified the V4 region of the 16S rRNA gene (Zeng et al., 2021).The PCR reaction was performed using the following procedures:Denaturation at 95 °C for 3 min, 27 cycles at 95 °C for 30 s, annealing at 55 °C for 30 s, extension at 72 °C for 45 s, and final elongation at 72 °C for 10 min. PCR was performed in a 20 μL mixture containing FastPfu polymerase (0.4 μL), 5 × FastPfu buffer (4 μL), 5 μM primers (0.8 μL), 2.5 mM dNTPs (2 μL) and template DNA (10 ng). After amplify-cation, PCR products were detected on 2% (w/v) agarose gels. The size of each amplicon was no less than 550 bp (Vaulot et al. 2022). High-throughput sequencing data were processed using the Deblur pipeline, which facilitated the alignment and denoising of raw reads, thereby generating bacterial amplicon sequence variant (ASV) tables for each study (Abbas-Egbariya et al. 2022). The raw sequence data have been submitted to the National Center for Biotechnology Information (NCBI) Sequence Read Archive (SRA) under BioProject accession number PRJNA899579 (SAMN31665566-SAMN31665571).

### Statistical analysis

Statistical analyses are conducted using the SPSS software package (Version 23; SPSS, Chicago, 1L), with one-way ANOVA employed to compare mean values between the different treatment groups CK and AMF. The neighbor joining phylogenetic tree is constructed using MEGA X (Kumar et al. 2018), with 1,000 bootstrap replicates, and visualized using iTOL version 4 (http://itol.embl.de) (Chen et al. 2020b; Xiao et al. 2025). Following screening, the RMT-based method (Faust et al. 2012) is utilized to construct microbial co-occurrence network with the first 2000 species in terms of richness http://ieg2.ou.edu/MENA (Chen et al. 2020c; Xiao et al. 2024), which is subsequently visualized using Gephi software. Nodes characterized by high intra-module connectivity (Zi) or inter-module connectivity (Pi) values (i.e., core microbiome) are designated as Keystone species. These include module hubs (Zi > 0.25, Pi ≤ 0.62), connectors (Zi ≤ 0.25, Pi > 0.62), and network hubs (Zi > 0.25, Pi > 0.62). To examine the correlations between microbial communities and environmental variables, Mantel tests are conducted using R software (version 4.0.2). Further examination of these differences is conducted using STAMP software (version 2.1.3) (Krzywinski et al. 2009), employing Welch's t-test to identify bacterial groups exhibiting significant differences. Additionally, volcano plots and bubble plots are generated utilizing the Hiplot online platform (https://hiplot.com.cn). Data are analyzed utilizing GraphPad Prism version 8.0.2, with results presented as mean ± standard deviation. Structural equation modeling (SEM) is performed using AMOS version 24 (IBM Corporation, NY, USA) (Chen et al. 2023; Chen et al. 2024a; Rahman et al. 2024).

## Results

### Physicochemical properties of tobacco and mycorrhizal colonization

Compared with CK treatment group, AMF inoculation significantly enhances soil spore counts and mycorrhizal infection rates (Table 1, *P* < 0.05). These findings suggested that AMF inoculation augments the presence and activity of beneficial soil microorganisms, which was essential for enhancing soil fertility and promoting plant growth. Furthermore, the aboveground and belowground biomass of AMF-inoculated plants were compared to that of the CK treatment group. Relative to the CK group, root length, plant height, root biomass, shoot biomass, and spore density increased by 0.73%, 0.75%, 12.90%, 1.61%, and 4.51%, respectively. These results indicated that AMF inoculation positively influences plant growth, leading to improved root and shoot development. Additionally, compared to the CK group, catalase (CAT) and superoxide dismutase (SOD) activities were significantly elevated in the AMF treatment group, along with increased levels of total nitrogen and organic carbon (*P* < 0.05).

**Table 1:** Growth of tobacco inoculated and uninoculated with AMF

Physicochemical parameter	Treatments	
	CK	AMF
Root length (cm)	20.07 ± 3.90b	34.80 ± 6.63a
Plant height (cm)	24.97 ± 5.95b	44.60 ± 1.48a
Root biomass (g plant ⁻^1^)	0.38 ± 0.19a	0.99 ± 0.47a
Shoot biomass (g plant ⁻^1^)	2.43 ± 0.62a	5.29 ± 1.79a
Spore density (10 g⁻^1^)	1.33 ± 0.58b	7.33 ± 0.58a
AMF colonization intensity (%)	4.77 ± 0.75b	65.10 ± 6.31a
AMF colonization rates (%)	13.33 ± 5.77b	96.67 ± 5.77a
TN (g/kg)	0.45 ± 0.005b	0.52 ± 0.005a
AP (mg/kg)	10.67 ± 0.58a	9.33 ± 0.58b
TOC (g/kg)	2.23 ± 0.06a	3.13 ± 0.15a
MDA (nmol/g)	4.49 ± 0.06a	2.56 ± 0.24b
CAT (U/g)	6.14 ± 0.52b	14.49 ± 1.31a
SOD (U/g)	106.92 ± 5.17b	158.11 ± 8.18a

Values were the means ± the standard error of the mean. Values in the same column followed by a lowercase letter indicate significant differences (*P* < 0.05)

Sample code abbreviations: *TN* total nitrogen, *AP* available phosphorus, *TOC* total organic carbon, *MDA* malondialdehyde, *CAT* catalase, *SOD* superoxide dismutase

### Composition and diversity of tobacco rhizosphere bacterial communities

**Fig. 1:**
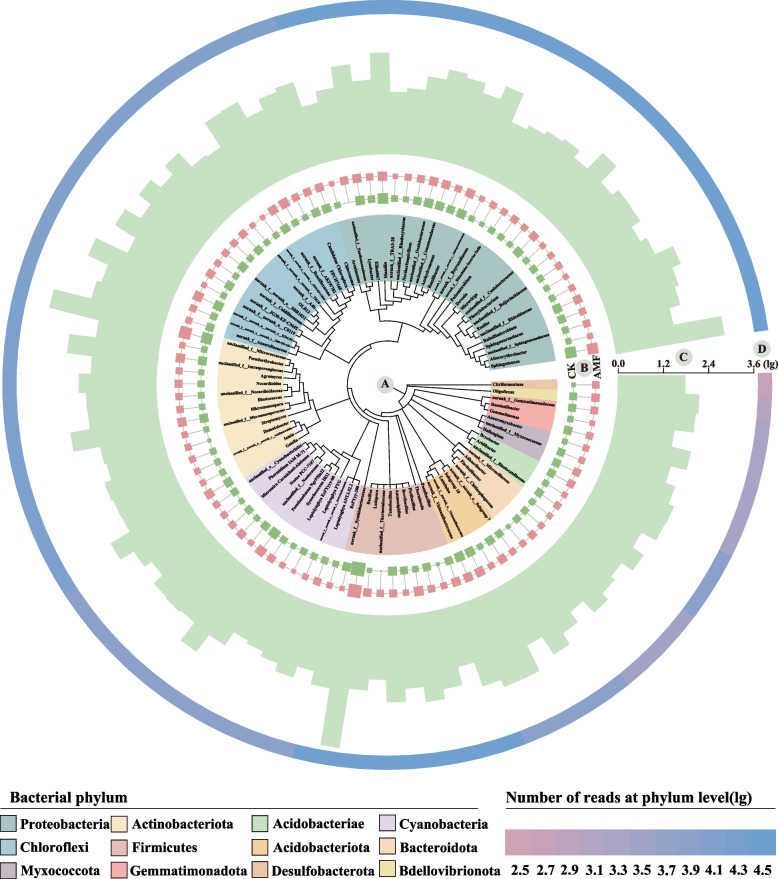
Phylogenetic tree of the rhizosphere bacteria genera in tobacco detected in the CK and AMF treatments. **a** Phylogenetic tree constructed by iTol tool and colored at the phylum level; **b** Number of reads in each genus for both treatments, different colors and quadrate sizes correspond to the different treatments and number of reads, respectively; **c** Number of reads in each genus for the sum of all samples; **d** Heatmap based on number of reads at phylum. The data are transformed using the natural logarithm (log^10^)

To explore the phylogenetic diversity and relationships within microbial communities, the MEGA software was employed for the construction of phylogenetic trees. The iTOL platform is subsequently utilized to visualize phylogenetic differences based on the abundance of the top 100 genera, which are designated as core microorganisms (Fig. 1). Analysis identified twelve bacterial phyla as core microorganisms. Notably, only a few branches exhibited bootstrap values below 60, suggesting that the phylogenetic tree demonstrated high reliability and robust intergeneric relationships. Among the bacteria, the majority of core microorganism genera were classified under the phyla Proteobacteria (28 genera), Chloroflexi (16 genera), Actinobacteriota (14 genera), and Myxococcota (12 genera), with the remaining genera distributed among the phyla Gemmatimonadetes, Acidobacteriae, Firmicutes, Desulfobacterota, Bacteroidota, and Bdellovibrionota.

**Fig. 2:**
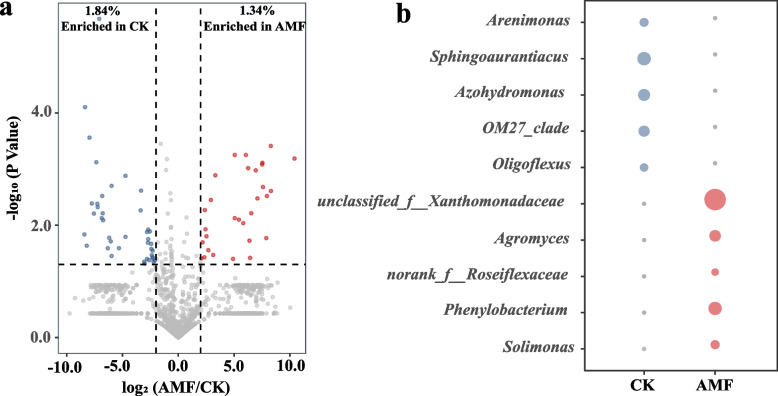
Differences and richness of different bacterial genera of tobacco rhizosphere bacteria community in different treatment groups. The results are shown as volcano plots (**a**) and bubble plots (**b**) which constructed by Hiplot. **a** Bacterial communities between different treatment groups with significantly increased or decreased at fixed thresholds (fold change > 4 and -log P value > 0.05) in the volcano plots. **b** The relative abundance of the 10 most significantly different bacterial genera is represented in the bubble plots, and the larger circles indicate higher richness

Volcano plots and bubble plots are generated to reveal differences in bacterial communities within tobacco rhizosphere soil across the two treatment groups (Fig. 2a, b). The results from the volcano plots indicate significant differences in the bacterial communities of rhizosphere soil between the two treatment groups at the genus level. In AMF-treated rhizosphere soil, specific bacterial genera made up about 1.34% of the total, compared to 1.84% in the CK group (Fig. 2a).

Analysis revealed 10 bacterial genera with the most significant differences in relative abundance between the two groups. In AMF group, the most abundant genera were *unclassified _ f _ _ Xanthomonadaceae, Agromyces, norank _ f _ _ Roseiflexaceae, Phenylobacterium* and *Solimonas*, while in CK group, *Arenimonas, Sphingoaurantiacus* and *Azohydromonas* are predominant (Fig. 2b).

### Co-occurrence networks of the ASVs in rhizosphere bacteria of tobacco

**Fig. 3:**
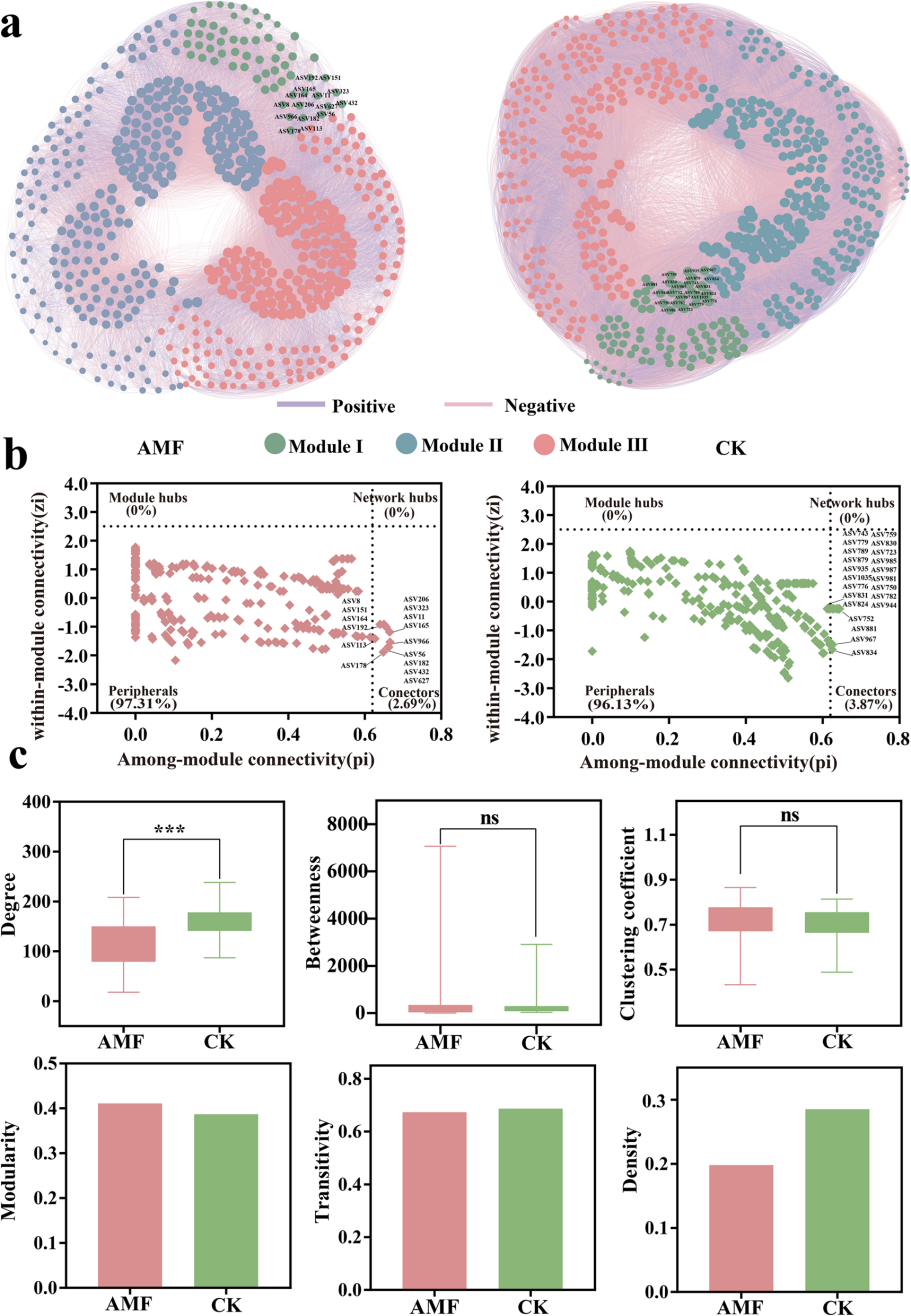
Co-occurrence network analysis reveals the interaction patterns of tobacco rhizosphere bacteria ASVs in two treatment groups. **a** The nodes are colored according to the module and the size of each node is proportional to the number of its connections. **b** The topological roles of the different ASVs shown in *Zi-Pi* plots. Threshold values of *Zi* and *Pi* for categorizing bacterial ASVs are 2.5 and 0.62, respectively. **c** Multiple network properties of microbial co-occurrence networks

Bacterial co-occurrence network relationships show distinct patterns between CK and AMF treatments (Fig. 3). Significant differences in topological properties, such as average clustering coefficient (avgCC), average connectivity (avgK), nodes, and edges, were observed across treatments. The CK network diagram had more nodes (567 vs. 556) and edges (45,736 vs. 30,494), indicating significantly higher values than those in the AMF network. Compared to the CK network diagram, avgCC (0.705 vs. 0.713) and avgK (161.326 vs. 109.691) were higher in the AMF network, suggesting that the AMF network enhances the complexity of the microbial symbiosis network. Additionally, the CK network exhibits a greater number of negative correlations compared to the AMF network (Fig. 3a).

In the CK network (Fig. 3b), 545 nodes (96.13% of the total node graph) are categorized as peripherals (Pi ≤ 0.62, Zi ≤ 2.5), indicating that the majority of nodes had low connectivity within modules and low topological significance. Conversely, the AMF network comprised 545 nodes (97.31% of the total node graph) classified as peripherals, which is a higher proportion than that observed in the CK network. The proportions of network connectors (defined as nodes connected to multiple modules with Pi ≥ 0.62) were 3.87% for CK and 2.69% for AMF network. Notably, neither module hubs nor network hubs were identified in either the CK or AMF networks due to the absence of nodes with Pi ≥ 0.62 and Zi ≥ 2.5. Despite the presence of outliers, node-level topological features (i.e., degree, betweenness, clustering coefficient, modularity and transitivity) were consistently higher in AMF networks compared to CK networks. This analysis indicates that arbuscular mycorrhizal fungi (AMF) are more frequently situated in central network positions (Fig. 3c, p < 0.001). These results suggested that AMF networks demonstrate a more intricate and interconnected architecture, with nodes more likely to occupy central positions within the network compared to CK networks.

### Functional composition spectrum of bacterial community

**Fig. 4:**
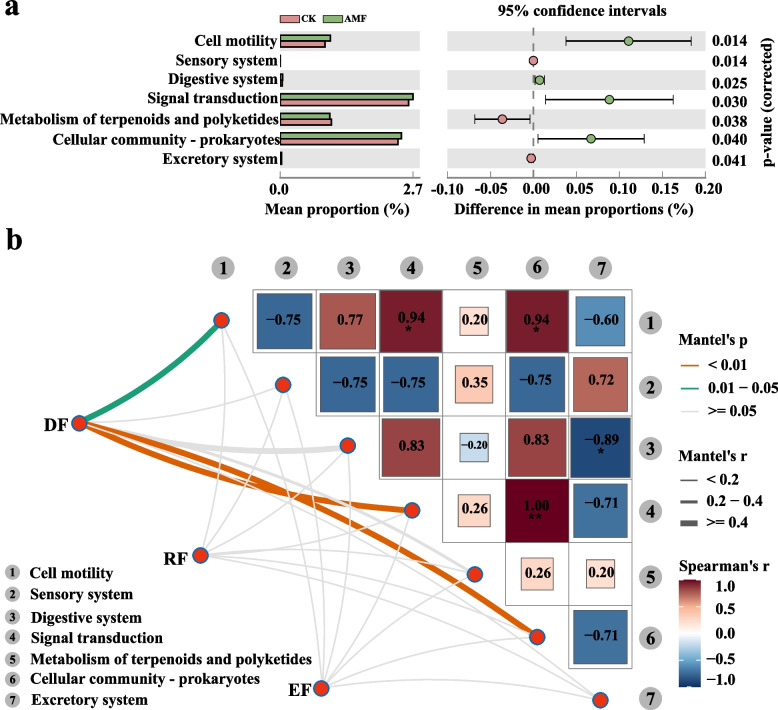
Between CK and AMF treatment groups significant difference analysis of tobacco rhizosphere bacteria. **a** Welch's *t*-test bar plot indicating the bacterial genera that are significantly different between AMF and CK. **b** Correlation analysis of three alpha diversities of bacterial with seven functions by Mantel tests. The width of the line corresponds Mantel's R statistic of distance correlation, and the color of the line indicates statistical significance. Pearson’s correlations between different environmental factors are indicated by shades of color

To further explore the functional implications of these network disparities, the Statistical Analysis of Metagenomic Profiles (STAMP) is utilized to compare the abundance of functional components between CK and AMF treatments (Fig. 4a). Seven representative functions are significantly influenced. For example, the addition of AMF significantly enhances the abundance of ASVs associated with metabolic functions, such as signal transduction and cell community-prokaryotes (Fig. 4a, P < 0.05).

To further examine the relationship between these functional profiles and the alpha diversity of tobacco rhizosphere bacterial communities, a correlation matrix is constructed to evaluate the associations between functional profiles and diversity indices (Shannon, Chao, and Simpson evenness) (Fig. 4b). The most robust positive correlations were observed among amino acid metabolism, carbohydrate metabolism, and the metabolism of cofactors and vitamins. Furthermore, signal transduction demonstrated a significant positive correlation with the prokaryotic cell community. The Mantel test indicated that several functional component profiles were strongly correlated with alpha diversity (Mantel’s *r* > 0.20, *P* < 0.05). The diversity of the bacterial community was closely linked to cell motility, signal transduction, and the prokaryotic cell community (Mantel’s *r* > 0.20, *P* < 0.05).

### The SEM revealed the influence of AMF on plants antioxidant capacity

**Fig. 5:**
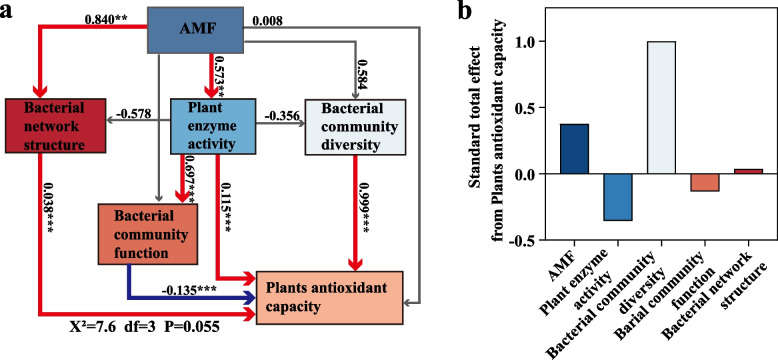
Structural equation modeling (SEM) reveals the connection among arbuscular mycorrhizal fungal (AMF), bacterial network structure, plant enzyme activity, bacterial community diversity, bacterial community function, plants antioxidant capacity in tobacco rhizosphere bacteria. **a** Red and blue lines represent positive and negative effects, respectively. And the gray lines represent non-significant relationships. Line width is proportional to relationship strength. The number next to the line segment is the standardized path coefficient. Significance levels are indicated: *P < 0.05, **P < 0.01, ***P < 0.001. **b** Standard total effect derived from SEM

Structural equation models (SEMs) are utilized to elucidate the direct and indirect effects of AMF, bacterial network structure, plant enzyme activity, bacterial community diversity, and function on plants antioxidant capacity within the tobacco rhizosphere bacterial community (Fig. 5a). The SEM analysis revealed that AMF had significant positive effects on bacterial network structure (λ = 0.840, *P* < 0.01) and soil enzyme activity (λ = 0.573, *P* < 0.01), whereas bacterial community diversity exhibited significant negative effects on plants antioxidant capacity. Plant enzyme activity had a significantly positive impact on both bacterial community function and plants antioxidant capacity (λ = 0.109, *P* < 0.05). Similarly, the diversity and functionality of the bacterial community within the tobacco rhizosphere exhibited significantly positive effects on the plants antioxidant capacity (λ = 0.152, *P* < 0.05). According to the standardized total effects derived from the SEM analysis, bacterial community diversity exerts a more pronounced influence on plants antioxidant capacity than AMF (Fig. 5b). Overall, bacterial community diversity is shown to have positive and direct effects on the plants antioxidant capacity.

## Discussion

### Effects of AMF on the growth of tobacco plants

The growth and metabolic activity of plants are primarily determined by their photosynthetic capacity and their ability to utilize soil nutrients. The presence of AMF can modify the plant's metabolic environment by affecting the availability of underground nutrients, thereby enhancing the plant's capacity to absorb and assimilate atmospheric CO₂. AMF could effectively mitigate oxidative stress by increasing the activities of antioxidant enzymes in host plants (Xiong et al., 2024). Our findings indicate that AMF inoculation led to a reduction in MDA content and an increase in CAT and SOD activities in tobacco (Table 1). In line with previous studies, the levels of antioxidant enzymes in inoculated plants were found to be elevated compared to those in uninoculated plants under various stress conditions (Zhou et al. 2024). Additionally, AMF inoculation significantly increased the activities of SOD, POD, and CAT in oat leaves relative to the control group (Wang et al. 2024). Notably, this study demonstrates a significant reduction in MDA content in tobacco plants subjected to AMF treatment (Table 1, *P* < 0.05). MDA levels are indicative of the extent of peroxidative damage experienced by plants under drought stress. Under conditions of low nutrient availability, membrane permeability increases, resulting in plant damage. Elevated MDA concentrations suggested severe damage to the cell membrane (Yu et al. 2024).

In the present study, AMF-inoculated seedlings exhibited increased biomass, with significant enhancements in plant height and fresh weight compared to uninoculated plants (Table 1, *P* < 0.05). This observation corroborates previous findings that report improved tobacco plant productivity following AMF inoculation (Khaliq et al., 2022). Furthermore, the colonization of AMF has been observed to augment abiotic stress tolerance in tomato crops (Chialva et al. 2024). This enhanced stress tolerance was intricately associated with improved nutrient acquisition mediated by mycorrhizal symbiosis. The AMF mycelial network extended beyond the root system, thereby increasing the surface area available for nutrient absorption. This expansion was particularly beneficial for phosphorus uptake, given the limited mobility of phosphorus in soil, which subsequently enhanced plant phosphorus nutrition and overall growth. Moreover, plants could stimulate hyphal branching by releasing specific rhizosphere signals, thus promoting the formation of mycorrhizal symbionts (Gong et al. 2022). AMF colonization significantly improved plant nutrient absorption. Following AMF inoculation, plants exhibited increased acquisition of both macro- and micronutrients, which in turn promotes the accumulation of photosynthetic components. In nutrient-deficient soils, AMF enhanced plant nutrient uptake by increasing the absorptive surface area of the host plant roots.

### Effect of AMF on the composition of tobacco bacterial communities

Extensive research has demonstrated that the populations of bacteria and actinomycetes in rhizosphere soil significantly increase following AMF inoculation, a finding that was corroborated by our experimental results (Liu et al. 2020). This phenomenon may be attributed to alterations in bacterial composition induced by AM fungi. Alternatively, it was possible that an increased flow of carbon into the soil food web occurs via AM mycelium (Janzen et al. 2024). Illumina MiSeq sequencing results indicate that the most dominant phyla within the tobacco rhizosphere bacterial community under AMF application are Proteobacteria, Chloroflexi, Actinobacteriota, and Myxococcota (Fig. 1). Notably, Proteobacteria were enriched at the phylum level in the AMF treatment and were recognized as one of the most diverse phyla within the bacterial community. The elevated concentration and diversity of soil resources might regulate competitive species interactions within this phylum, thereby enhancing its abundance (Xiao et al. 2024). Furthermore, Proteobacteria played a crucial role as key regulators in global carbon, nitrogen, and sulfur cycles (Huang et al. 2023). Research has demonstrated that nitrogen-fixing bacteria within Proteobacteria contribute to nitrification, enhancing soil nutrients (Li et al. 2021; Fu et al. 2022). Bacteroides, Proteobacteria, and Actinomycetes have been shown to aid soil protease production. In this study, AMF addition notably increased the relative abundance of Proteobacteria in the tobacco rhizosphere bacterial community. Chloroflexi, strictly anaerobic bacteria, were expected to be minimally present in these conditions. Known for utilizing complex carbohydrates and peptides, Chloroflexi form multicellular filaments that degrade microbial products (Dang et al. 2024; Hashimi and Tocheva 2024). Thus, in this study, Chloroflexi might metabolize fungal organic deposits, enhancing their presence in the mycorrhizal rhizosphere (Rodríguez-Caballero et al. 2017). Actinomycetes enhanced plant phosphorus uptake through dissolution or mineralization and promote nitrogen fixation symbiosis. They also produced extracellular metabolites that suppress pathogens and sometimes act as growth regulators (Vurukonda et al. 2018). Myxococcota, as predators in the mycelial food web, consume carbon-rich compounds transported by AMF from the plant host, contributing significantly to the tobacco rhizosphere (Nuccio et al. 2022). Bacteria enriched under AMF treatment aid in nutrient acquisition, improve tolerance to low-nutrient conditions, and support plant growth. Thus, AMF addition enhances the diversity and stability of the soil rhizosphere bacterial community, positively impacting plant growth.

### Effects of AMF on the structural diversity and function of tobacco bacterial communities

An ecological co-occurrence network encompasses a variety of interspecific interactions, wherein species were linked through diverse positive (e.g., mutualism and commensalism) and negative (e.g., competition and predation) relationships (Chen et al. 2021; Li et al. 2024). The results of network analysis reveal that the AMF treatment exhibits a greater number of nodes compared to the CK treatment (Fig. 3). This phenomenon might be attributed to the ability of AMF to enrich a greater number of core soil microorganisms, thereby enhancing network partitioning or modularization. It has been documented that highly modular networks contribute to the stability of microbial communities and enable their swift response to environmental changes (Wu et al. 2024). Generally, increased modularity enhanced the probability of identifying more keystone groups, which were crucial for maintaining complex interactions within the co-occurrence network and for supporting biogeochemical processes across diverse environments (Wei et al. 2024). In alignment with our findings, AMF shows to recruit specific key bacterial taxa, including 4 ASVs from Proteobacteria, 3 from Actinobacteriota, 2 from Firmicutes, 2 from Cyanobacteria, 2 from Chloroflexi, and 1 from Bacteroidota (Fig. 3). Proteobacteria and Actinobacteriota within AMF demonstrated a significant association with AMF inoculation, exhibiting heightened sensitivity to it (Liu et al. 2023a, b). Proteobacteria constituted the predominant phylum within the rhizosphere microbial community, playing a crucial role in regulating metabolic activities and collaborating with other microorganisms to enhance plant stress resistance and maintain the stability of the microbial community network (Ling et al. 2022). The increased abundance of Actinobacteriota enhances its synergistic interactions with other microorganisms, thereby augmenting the overall functionality of the tobacco rhizosphere microbial community (Ren et al. 2024). This enhancement facilitates more efficient nutrient and water acquisition by tobacco plants, improves their stress resistance and adaptability, and promotes plant growth. Notably, compared to the CK network, the AMF-treated symbiotic network exhibits higher average connectivity (avgK) and average clustering coefficient (avgCC), suggesting that the AMF-treated network possesses a more complex microbial community structure characterized by closer and more interconnected nodes. Elevated average connectivity leaded to the formation of highly interconnected networks, thereby increasing resistance to environmental changes (Chen et al. 2020a). A greater number of positive interactions indicates enhanced cooperation and robust syntrophic relationships within complex microbial communities (Xiong et al. 2017). This suggests that the ecological stability of the community in the tobacco rhizosphere is heightened, potentially facilitating better adaptation to environmental changes. Increased interactions among AMF species might enhance their functionality, such as their roles in nutrient cycling and promoting plant growth (Wang et al. 2021; Duan et al. 2024).

Considering this information, significant differences in soil microbial community structure and function were observed between control (CK) and AMF treatments. This study further explored the relationships among AMF, bacterial network structure, soil enzyme activity, bacterial community diversity, bacterial community function, and plant antioxidant activity within tobacco rhizosphere soil (Fig. 5). SEM, an a priori method, was employed to visualize causal relationships between key variables by fitting data to a causal hypothesis model. (Chen et al. 2021; Gao et al. 2024). This study concentrated on critical environmental parameters, specifically AMF and soil bacterial communities, which have been documented in several studies to significantly influence plants antioxidant capacity (Gao 2024; Zhang et al. 2024).

The findings corroborated that AMF was the most substantial positive contributor to plant enzyme activity, aligning with previous research (Jerbi et al. 2022). This positive contribution is closely linked to the protective effects of AMF against oxidative damage. AMF colonization mitigates the detrimental effects of ROS, shields plants from oxidative damage, and ultimately fosters the growth of tobacco plants. The presence of AM fungi was found to reduce membrane lipid peroxidation. Increased membrane lipid peroxidation enhanced membrane permeability, leads to electrolyte leakage, and ultimately compromises the integrity of the cell membrane system (Yuan et al. 2024). In summary, AMF inoculation enhances plant growth and photosynthesis by upregulating the plant's antioxidant systems.

## Conclusions

In conclusion, the structure of the bacterial community within the tobacco rhizosphere is significantly influenced by AMF. Post-AMF inoculation, there is a notable enhancement in both the richness and diversity of the bacterial community in the tobacco rhizosphere, accompanied by an enrichment of bacteria that promote plant growth. The co-occurrence network of the rhizosphere bacterial community exhibits increased interconnectivity and complexity, as evidenced by higher average connectivity and modularity. Furthermore, SEM demonstrates that AMF positively modulate the plants antioxidant capacity by influencing the rhizosphere bacterial community. These findings underscore the advantages of AMF inoculation in augmenting the bacterial communities of the tobacco rhizosphere under nutrient-deficient conditions and elucidate the critical role of AMF in enhancing plants antioxidant capacity through the regulation of rhizosphere bacterial assemblages.

## Data Availability

The data supporting the findings of this study are available from the authors upon reasonable request.

## References

[CR1] Abbas-Egbariya H, Haberman Y, Braun T, Hadar R, Denson L, Gal-Mor O, Amir A. Meta-analysis defines predominant shared microbial responses in various diseases and a specific inflammatory bowel disease signal. Genome Biol. 2022;23:61. 10.1186/s13059-022-02637-7. 35197084 10.1186/s13059-022-02637-7 PMC8867743

[CR2] Abkenar MB, Mozafari H, Karimzadeh K, Rajabzadeh F, Azimi R. Arbuscular Mycorrhizal Fungi (AMF) and Plant Growth-promoting Rhizobacteria (PGPR) as an Alternative to Mineral Fertilizers to Improve the Growth, Essential Oil Profile, and Phenolic Content of Satureja Macrantha L. J Crop Health. 2024;76:347–56. 10.1007/s10343-023-00934-0.

[CR3] Afridi MS, Fakhar A, Kumar A, Ali S, Medeiros FH, Muneer MA, Ali H, Saleem M. Harnessing microbial multitrophic interactions for rhizosphere microbiome engineering. Microbiol Res. 2022;127199. 10.1016/j.micres.2022.127199 10.1016/j.micres.2022.127199 36137486

[CR4] Alsherif EA, Almaghrabi O, Elazzazy AM, Abdel-Mawgoud M, Beemster GT, AbdElgawad H. Carbon nanoparticles improve the effect of compost and arbuscular mycorrhizal fungi in drought-stressed corn cultivation. Plant Physiol Biochem. 2023;194:29–40. 10.1016/j.plaphy.2022.11.005. 36371897 10.1016/j.plaphy.2022.11.005

[CR5] Bilgili A, Bilgili AV. Comparison of compost, PGPR, and AMF in the biological control of tomato Fusarium wilt disease. Eur J Plant Pathol. 2023;167:771–86. 10.1007/s10658-023-02710-2.

[CR6] Cao M, Xiang Y, Huang L, Li M, Jin C, He C, Xin G. Winter forage crops influence soil properties through establishing different arbuscular mycorrhizal fungi communities in paddy field. Adv Biotechnol. 2024;2:30. 10.1007/s44307-024-00037-5. 10.1007/s44307-024-00037-5 PMC11740874 39883251

[CR7] Chen J, Cui Y, Xiao Q, Lin K, Wang B, Zhou J, Li X. Difference in microbial community structure along a gradient of crater altitude: insights from the Nushan volcano. Appl Environ Microbiol. 2024a;90(8):e00753–24. 10.1128/aem.00753-24. 10.1128/aem.00753-24 PMC11337807 39028194

[CR8] Chen J, Guo Y, Li F, Zheng Y, Xu D, Liu H, Liu X, Wang X, Bao Y. Exploring the effects of volcanic eruption disturbances on the soil microbial communities in the montane meadow steppe. Environ Pollut. 2020a;267: 115600. 10.1016/j.envpol.2020.115600. 33254629 10.1016/j.envpol.2020.115600

[CR9] Chen J, Li Z, Xu D, Xiao Q, Liu H, Li X, Chao L, Qu H, Zheng Y, Liu X, Wang P, Bao Y. Patterns and drivers of microbiome in different rock surface soil under the volcanic extreme environment. iMeta. 2023;2(3):e122. 10.1002/imt2.122. 10.1002/imt2.122 PMC10989942 38867933

[CR10] Chen J, Lin K, Huang T, Geng X, Li Z, Wang B, Xiao Q, Li X. The active effect of Rhizophagus irregularis inoculants on maize endophytic bacteria community. iMetaOmics. 2024b;1(1):e23. 10.1002/imo2.23. 10.1002/imo2.23 PMC12806334 41675541

[CR11] Chen J, Nan J, Xu D, Mo L, Zheng Y, Chao L, Qu H, Guo Y, Li F, Bao Y. Response differences between soil fungal and bacterial communities under opencast coal mining disturbance conditions. CATENA. 2020b;194: 104779. 10.1016/j.catena.2020.104779.

[CR12] Chen J, Xu D, Chao L, Liu H, Bao Y. Microbial assemblages associated with the rhizosphere and endosphere of an herbage. Leymus Chinensis Microb Biotechnol. 2020c;13:1390–402. 10.1111/1751-7915.13558. 32227622 10.1111/1751-7915.13558 PMC7415361

[CR13] Chen J, Xu D, Zheng Y, Chao L, Liu H, Qu H, Wang B, Li F, Guo Y, Bao Y. Distinct effects of volcanic cone types on soil microbiomes: Evidence from cinder cone and spatter cone. CATENA. 2021;200: 105180. 10.1016/j.catena.2021.105180.

[CR14] Chialva M, Stelluti S, Novero M, Masson S, Bonfante P, Lanfranco L. Genetic and functional traits limit the success of colonisation by arbuscular mycorrhizal fungi in a tomato wild relative. Plant Cell Environ. 2024;47:4275–92. 10.1111/pce.15007. 38953693 10.1111/pce.15007

[CR15] Cui H, Chen P, He C, Jiang Z, Lan R, Yang J. Soil microbial community structure dynamics shape the rhizosphere priming effect patterns in the paddy soil. Sci Total Environ. 2023;857: 159459. 10.1016/j.scitotenv.2022.159459. 36252670 10.1016/j.scitotenv.2022.159459

[CR16] Dang YR, Cha QQ, Liu SS, Wang SY, Li PY, Li CY, Wang P, Chen XL, Tian JW, Xin Y, Chen Y, Zhang YZ, Qin QL. Phytoplankton-derived polysaccharides and microbial peptidoglycans are key nutrients for deep-sea microbes in the Mariana Trench. Microbiome. 2024;12:77. 10.1186/s40168-024-01789-x. 38664737 10.1186/s40168-024-01789-x PMC11044484

[CR17] Ding Y, Gao X, Shu D, Siddique KH, Song X, Wu P, Li C, Zhao X. Enhancing soil health and nutrient cycling through soil amendments: Improving the synergy of bacteria and fungi. Sci Total Environ. 2024;923: 171332. 10.1016/j.scitotenv.2024.171332. 38447716 10.1016/j.scitotenv.2024.171332

[CR18] Duan S, Feng G, Limpens E, Bonfante P, Xie X, Zhang L. Cross-kingdom nutrient exchange in the plant–arbuscular mycorrhizal fungus–bacterium continuum. Nat Rev Microbiol. 2024;22:773–90. 10.1038/s41579-024-01073-7. 39014094 10.1038/s41579-024-01073-7

[CR19] Edwards J, Johnson C, Santos-Medellín C, Lurie E, Podishetty NK, Bhatnagar S, Eisen JA, Sundaresan V. Structure, variation, and assembly of the rootassociated microbiomes of rice. Proc Nat Acad Sci. 2015;112(8):E911–20. 10.1073/pnas.1414592112. 10.1073/pnas.1414592112 PMC4345613 25605935

[CR20] Faust K, Sathirapongsasuti JF, Izard J, Segata N, Gevers D, Raes J, Huttenhower C. Microbial co-occurrence relationships in the human microbiome. PLoS Comput Biol. 2012;8:e1002606. 10.1371/journal.pcbi.1002606. 22807668 10.1371/journal.pcbi.1002606 PMC3395616

[CR21] Fu W, Chen X, Zheng X, Liu A, Wang W, Ji J, Wang G, Guan C. Phytoremediation potential, antioxidant response, photosynthetic behavior and rhizosphere bacterial community adaptation of tobacco (Nicotiana tabacum L.) in a bisphenol A-contaminated soil. Environ Sci Pollut Res. 2022;29:84366–84382. 10.1007/s11356-022-21765-y 10.1007/s11356-022-21765-y 35780263

[CR22] Gao JF. Experimental guidance for plant physiology. Beijing, China: China Higher Education Press; 2006.

[CR23] Gao M, Zhang Q, Wu S, Wu L, Cao P, Zhang Y, Rong L, Fang B, Yuan C, Yao Y, Wang Y, Sun H. Contamination status of novel organophosphate esters derived from organophosphite antioxidants in soil and the effects on soil bacterial communities. Environ Sci Technol. 2024;58:10740–51. 10.1021/acs.est.3c10611. 38771797 10.1021/acs.est.3c10611

[CR24] Gong J, Zheng Z, Zheng B, Liu Y, Hu R, Gong J, Li S, Tian L, Tian X, Li J, Rang Z. Deep tillage reduces the dependence of tobacco (Nicotiana tabacum L.) on arbuscular mycorrhizal fungi and promotes the growth of tobacco in dryland farming. Can J Microbiol. 2022;68:203–213. 10.1139/cjm-2021-0272 10.1139/cjm-2021-0272 35007166

[CR25] Hashimi A, Tocheva EI. Cell envelope diversity and evolution across the bacterial tree of life. Nat Microbiol. 2024;9:2475–87. 10.1038/s41564-024-01812-9. 39294462 10.1038/s41564-024-01812-9

[CR26] Huang J, Liu X, Liu J, Zhang Z, Zhang W, Qi Y, Li W, Chen Y. Changes of soil bacterial community, network structure, and carbon, nitrogen and sulfur functional genes under different land use types. Catena. 2023;231:107385. 10.1016/j.catena.2023.107385.

[CR27] Janzen HH. RUSSELL REVIEW Soil carbon stewardship: Thinking in circles. European J Soil Sci. 2024;75: e13536. 10.1111/ejss.13536.

[CR28] Jerbi M, Labidi S, Laruelle F, Tisserant B, Dalpé Y, Lounès-Hadj Sahraoui A, Ben Jeddi F. Contribution of native and exotic arbuscular mycorrhizal fungi in improving the physiological and biochemical response of hulless barley (Hordeum vulgare ssp. nudum L.) to drought. J Soil Sci Plant Nutr. 2022;22:2187–2204. 10.1007/s42729-022-00802-2

[CR29] Jiang C, Kong D, Li Y, Sun J, Chen Z, Yang M, Cao S, Yu C, Wang Z, Jiang J, Zhu C, Zhang N, Sun G, Zhang Q. Degradation and mechanism analysis of protein macromolecules by functional bacteria in tobacco leaves. Front Microbio. 2024;15:1416734. 10.3389/fmicb.2024.1416734. 10.3389/fmicb.2024.1416734 PMC11258012 39035444

[CR30] Khaliq A, Perveen S, Alamer KH, Zia Ul HaqM, Rafique Z, Alsudays IM, Althobaiti AT, Saleh MA, Hussain S, Attia H. Arbuscular Mycorrhizal Fungi Symbiosis to Enhance Plant–Soil Interaction. Sustainability. 2022;14:7840. 10.3390/su14137840

[CR31] Krzywinski M, Schein J, Birol I, Connors J, Gascoyne R, Horsman D, Jones SJ, Marra MA. Circos: an information aesthetic for comparative genomics. Genome Res. 2009;19:1639–45. 10.1101/gr.092759.109. 19541911 10.1101/gr.092759.109 PMC2752132

[CR32] Kumar S, Stecher G, Li M, Knyaz C, Tamura K. MEGA X: Molecular Evolutionary Genetics Analysis across Computing Platforms. Mol Biol Evol. 2018;35:1547–9. 10.1093/molbev/msy096. 29722887 10.1093/molbev/msy096 PMC5967553

[CR33] Li J, Cai B, Chang S, Yang Y, Zi S, Liu T. Mechanisms associated with the synergistic induction of resistance to tobacco black shank in tobacco by arbuscular mycorrhizal fungi and β-aminobutyric acid. Front Plant Sci. 2023a;14:1195932. 10.3389/fpls.2023.1195932. 37434599 10.3389/fpls.2023.1195932 PMC10330952

[CR34] Li J, Ye X, Zhang Y, Chen J, Yu N, Zou H. Maize straw deep-burying promotes soil bacteria community abundance and improves soil fertility. J Soil Sci Plant Nutr. 2021;21:1397–407. 10.1007/s42729-021-00448-6.

[CR35] Li L, Liu Q, Ge S, Tang M, He L, Zou Y, Yu J, Zhou Y. SlIAA23-SlARF6 module controls arbuscular mycorrhizal symbiosis by regulating strigolactone biosynthesis in tomato. Plant Cell Environ. 2023b;46:1921–34. 10.1111/pce.14580. 36891914 10.1111/pce.14580

[CR36] Li P, Tian Y, Yang K, Tian M, Zhu Y, Chen X, Hu R, Qin T, Liu Y, Peng S, Zhen Y, Liu Z, Ao H, Li J. Mechanism of microbial action of the inoculated nitrogen-fixing bacterium for growth promotion and yield enhancement in rice (Oryza sativa L.). Adv Biotechnol. 2024;2:32. 10.1007/s44307-024-00038-4 10.1007/s44307-024-00038-4 PMC11709144 39883349

[CR37] Li Z, Li Y, Zhang Q, Zhang Z, Jiang J, Huang T, Mei C, Wu F, Cheng B, Cheng B, Chen J. Synergistic mechanisms of AMF and biochar driving rhizosphere fungal community in shallot in barren soil. Hortic Plant J. 2024;10(5):1252–6. 10.1016/j.hpj.2024.01.010.

[CR38] Ling N, Wang T, Kuzyakov Y. Rhizosphere bacteriome structure and functions. Nat Commun. 2022;13:836. 10.1038/s41467-022-28448-9. 35149704 10.1038/s41467-022-28448-9 PMC8837802

[CR39] Liu Q, Cheng L, Nian H, Jin J, Lian T. Linking plant functional genes to rhizosphere microbes: a review. Plant Biotechnol J. 2023a;21:902–17. 10.1111/pbi.13950. 36271765 10.1111/pbi.13950 PMC10106864

[CR40] Liu H, Zhang J, Zhang L, Zhang X, Yang R. Funneliformis mosseae influences leaf decomposition by altering microbial communities under saline-alkali conditions. Sci Total Environ. 2023b;895: 165079. 10.1016/j.scitotenv.2023.165079. 37356763 10.1016/j.scitotenv.2023.165079

[CR41] Liu N, Shao C, Sun H, Liu Z, Guan Y, Wu L, Zhang L, Pan X, Zhang Z, Zhang Y. Arbuscular mycorrhizal fungi biofertilizer improves American ginseng (Panax quinquefolius L.) growth under the continuous cropping regime. Geoderma. 2020;363:114155. 10.1016/j.geoderma.2019.114155

[CR42] Liu N, Zhao J, Du J, Hou C, Zhou X, Chen J, Zhang Y. Non-phytoremediation and phytoremediation technologies of integrated remediation for water and soil heavy metal pollution: A comprehensive review. Sci Total Environ. 2024;174237. 10.1016/j.scitotenv.2024.174237 10.1016/j.scitotenv.2024.174237 38942300

[CR43] Nuccio EE, Blazewicz SJ, Lafle M, Campbell AN, Kakouridis A, Kimbrel JA, Wollard J, Vyshenska D, Riley R, Tomatsu A. HT-SIP: a semi-automated stable isotope probing pipeline identifies cross-kingdom interactions in the hyphosphere of arbuscular mycorrhizal fungi. Microbiome. 2022;10:199. 10.1186/s40168-022-01391-z. 36434737 10.1186/s40168-022-01391-z PMC9700909

[CR44] Rahman MA, Tohan MM, Zaman S, Islam MA, Rahman MS, Howlader MH, Kundu S. A structural equation modelling to explore the determinants of mental health disorders among reproductive-aged women in Nepal: a nation-wide cross-sectional survey. BMC Psychiatry. 2024;24:867. 10.1186/s12888-024-06249-2. 39617912 10.1186/s12888-024-06249-2 PMC11610098

[CR45] Ren K, Yang X, Li J, Ji H, Gu K, Chen Y, Liu M, Luo Y, Jiang Y. Alleviating the adverse effects of Cd–Pb contamination through the application of silicon fertilizer: Enhancing soil microbial diversity and mitigating heavy metal contamination. Chemosphere. 2024;352: 141414. 10.1016/j.scitotenv.2023.167409. 38336042 10.1016/j.chemosphere.2024.141414

[CR46] Riaz M, Kamran M, Fang Y, Wang Q, Cao H, Yang G, Deng L, Wang Y, Zhou Y, Anastopoulos I, Xiurong W. Arbuscular mycorrhizal fungi-induced mitigation of heavy metal phytotoxicity in metal contaminated soils: A critical review. J Hazard Mater. 2021;402: 123919. 10.1016/j.jhazmat.2020.123919. 33254825 10.1016/j.jhazmat.2020.123919

[CR47] Rodríguez-Caballero G, Caravaca F, Fernández-González AJ, Alguacil M, Fernández-López M, Roldán A. Arbuscular mycorrhizal fungi inoculation mediated changes in rhizosphere bacterial community structure while promoting revegetation in a semiarid ecosystem. Sci Total Environ. 2017;584:838–48. 10.1016/j.scitotenv.2017.01.128. 28131451 10.1016/j.scitotenv.2017.01.128

[CR48] Timofeeva AM, Galyamova MR, Sedykh SE. How do plant growth-promoting bacteria use plant hormones to regulate stress reactions? Plants. 2024;13:2371. 10.3390/plants13172371. 39273855 10.3390/plants13172371 PMC11397614

[CR49] Vaulot D, Geisen S, Mahé F, Bass D. pr2-primers: An 18S rRNA primer database for protists. Mol Ecol Resour. 2022;22:168–79. 10.1111/1755-0998.13465. 34251760 10.1111/1755-0998.13465

[CR50] Vurukonda SSKP, Giovanardi D, Stefani E. 2018. Plant growth promoting and biocontrol activity of Streptomyces spp. as endophytes. Int J Mol Sci. 2018;19:952. 10.3390/ijms19040952 10.3390/ijms19040952 PMC5979581 29565834

[CR51] Wang C, Zheng MM, Song WF, Chen RF, Zhao XQ, Wen SL, Zheng ZS, Shen RF. Biogeographic patterns and co-occurrence networks of diazotrophic and arbuscular mycorrhizal fungal communities in the acidic soil ecosystem of southern China. Appl Soil Ecol. 2021;158: 103798. 10.1016/j.apsoil.2020.103798.

[CR52] Wang Z, Lian J, Liang J, Wei H, Chen H, Hu W, Tang M. Arbuscular mycorrhizal symbiosis modulates nitrogen uptake and assimilation to enhance drought tolerance of Populus cathayana. Plant Physiol Biochem. 2024;210: 108648. 10.1016/j.plaphy.2024.108648. 38653094 10.1016/j.plaphy.2024.108648

[CR53] Wang Z, Tang J, Zhu L, Feng Y, Yue L, Wang C, Xiao Z, Chen, F. (2022). Nanomaterial-induced modulation of hormonal pathways enhances plant cell growth. Environ. Sci.: Nano.2022;9:1578–1590. 10.1039/D2EN00251E

[CR54] Wei J, Chen W, Wen D. Rare biosphere drives deterministic community assembly, co-occurrence network stability, and system performance in industrial wastewater treatment system. Environ Int. 2024;190: 108887. 10.1016/j.envint.2024.108887. 39024826 10.1016/j.envint.2024.108887

[CR55] Wu H, Gao T, Hu A, Wang J. Network complexity and stability of microbes enhanced by microplastic diversity. Environ Sci Technol. 2024;58:4334–45. 10.1021/acs.est.3c08704. 38382548 10.1021/acs.est.3c08704

[CR56] Xiao D, He X, Zhang W, Chen M, Hu P, Wu H, Liao X, Wang K. Strengthen interactions among fungal and protistan taxa by increasing root biomass and soil nutrient in the topsoil than in the soil-rock mixing layer. J Environ Manage. 2024;355: 120468. 10.1016/j.jenvman.2024.120468. 38430883 10.1016/j.jenvman.2024.120468

[CR57] Xiao Q, Wang B, Cui Y, Li Z, Geng X, Lin K, Li X, Chen J. Distinct co-occurrence patterns and assembly processes of abundant and rare taxa under cadmium stress in volcanic areas. Catena. 2025;248:108604. 10.1016/j.catena.2024.108604.

[CR58] Xiao Q, Wang B, Li Z, Zhang Z, Xie K, Zhou J, Lin K, Geng X, Li C, Chen J. The assembly process and co-occurrence network of soil microbial community driven by cadmium in volcanic ecosystem. Resour Environ Sustain. 2024;17:100164. 10.1016/j.resenv.2024.100164.

[CR59] Xiong W, Li R, Ren Y, Liu C, Zhao Q, Wu H, Jousset A, Shen Q. Distinct roles for soil fungal and bacterial communities associated with the suppression of vanilla Fusarium wilt disease. Soil Biol Biochem. 2017;107:198–207. 10.1016/j.soilbio.2017.01.010.

[CR60] Xiong X, Wei YQ, Liu MH, Liu N, Zhang YJ. Localized and systemic abilities of arbuscular mycorrhizal fungi to control growth, antioxidant defenses, and the nutrient uptake of alfalfa under uniform and non-uniform salt stress. Plant Soil. 2024;1–19. 10.1007/s11104-024-07008-8

[CR61] Yu L, Zhou Y, Chen Y, Wan, Y, Gu Q, Song D. Antifungal activity and mechanism of Litsea cubeba (Lour.) Persoon essential oil against the waxberry spoilage fungi Penicillium oxalicum and its potential application. Int J Food Microbiol. 2024;411:110512. 10.1016/j.ijfoodmicro.2023.110512 10.1016/j.ijfoodmicro.2023.110512 38043475

[CR62] Yuan Q, Jiang Y, Yang Q, Li W, Gan G, Cai L, Li W, Qin C, Yu C, Wang Y. Mechanisms and control measures of low temperature storage-induced chilling injury to solanaceous vegetables and fruits. Front Plant Sci. 2024;2024(15):1488666. 10.3389/fpls.2024.1488666. 10.3389/fpls.2024.1488666 PMC11586204 39588087

[CR63] Zeng Q, An S. Identifying the biogeographic patterns of rare and abundant bacterial communities using different primer sets on the loess plateau. Microorganisms. 2021;9:139. 10.3390/microorganis-ms9010139. 33435426 10.3390/microorganisms9010139 PMC7827256

[CR64] Zhang J, Deng Y, Ge X, Shi X, Fan X, Dong K, Chen L, Zhao N, Gao Y, Ren A. The beneficial effect of Epichloë endophytes on the growth of host grasses was affected by arbuscular mycorrhizal fungi, pathogenic fungi and nitrogen addition. Environ Exp Bot. 2022a;201:104979. 10.1016/j.envexpbot.2022.105089.

[CR65] Zhang H, Sun X, Dai M. Improving crop drought resistance with plant growth regulators and rhizobacteria: Mechanisms, applications, and perspectives. Plant Commun. 2022b;3: 100228. 10.1016/j.xplc.2021.100228. 35059626 10.1016/j.xplc.2021.100228 PMC8760038

[CR66] Zhang R, Yang W, Pan Q, Zeng Q, Yan C, Bai X, Liu Y, Zhang L, Li B. Effects of long-term blue light irradiation on carotenoid biosynthesis and antioxidant activities in Chinese cabbage (Brassica rapa L. ssp. pekinensis). Food Res Int. 2023;174:113661. 10.1016/j.foodres.2023.113661 10.1016/j.foodres.2023.113661 37981380

[CR67] Zhang Z, Zhao L, Jin Q, Luo Q, He H. Combined contamination of microplastic and antibiotic alters the composition of microbial community and metabolism in wheat and maize rhizosphere soil. J Hazard Mater. 2024;473: 134618. 10.1016/j.jhazmat.2024.134618. 38761764 10.1016/j.jhazmat.2024.134618

[CR68] Zhao M, Zheng X, Su Z, Shen G, Xu Y, Feng Z, Li W, Zhang S, Cao G, Zhang J, Wu J. MicroRNA399s and strigolactones mediate systemic phosphate signaling between dodder-connected host plants and control association of host plants with rhizosphere microbes. New Phytol. 2025;245:1263–76. 10.1111/nph.20266. 39555671 10.1111/nph.20266

[CR69] Zhou M, Li H, Xi L, Shi F, Li X, Wang F, Liu X, Su Y, Wei Y. Influence of rhizospheric symbiotic microorganisms on the behavioural effects of antimony in soil-plant system: insights from a proteomic perspective. J Hazard Mater. 2024;480:136328. 10.1016/j.jhazmat.2024.136328. 39476691 10.1016/j.jhazmat.2024.136328

